# Management of irritable bowel syndrome in primary care: feasibility randomised controlled trial of mebeverine, methylcellulose, placebo and a patient self-management cognitive behavioural therapy website. (MIBS trial)

**DOI:** 10.1186/1471-230X-10-136

**Published:** 2010-11-18

**Authors:** Hazel A Everitt, Rona E Moss-Morris, Alice Sibelli, Laura Tapp, Nicholas S Coleman, Lucy Yardley, Peter W Smith, Paul S Little

**Affiliations:** 1 Primary Medical Care, School of Medicine, University of Southampton, Southampton SO17 1BJ, UK; 2 School of Psychology, University of Southampton, Southampton SO17 1BJ, UK; 3 Southampton University Hospital Trust, Southampton, UK; 4 Southampton Statistical Sciences Research Institute, University of Southampton, Southampton SO17 1BJ, UK

## Abstract

**Background:**

IBS affects 10-22% of the UK population. Abdominal pain, bloating and altered bowel habit affect quality of life, social functioning and time off work. Current GP treatment relies on a positive diagnosis, reassurance, lifestyle advice and drug therapies, but many suffer ongoing symptoms.

A recent Cochrane review highlighted the lack of research evidence for IBS drugs. Neither GPs, nor patients have good evidence to inform prescribing decisions. However, IBS drugs are widely used: In 2005 the NHS costs were nearly £10 million for mebeverine and over £8 million for fibre-based bulking agents. CBT and self-management can be helpful, but poor availability in the NHS restricts their use. We have developed a web-based CBT self-management programme, Regul8, based on an existing evidence based self-management manual and in partnership with patients. This could increase access with minimal increased costs.

**Methods/Design:**

The aim is to undertake a feasibility factorial RCT to assess the effectiveness of the commonly prescribed medications in UK general practice for IBS: mebeverine (anti-spasmodic) and methylcellulose (bulking-agent) and Regul8, the CBT based self-management website.

135 patients aged 16 to 60 years with IBS symptoms fulfilling Rome III criteria, recruited via GP practices, will be randomised to 1 of 3 levels of the drug condition: mebeverine, methylcellulose or placebo for 6 weeks and to 1 of 3 levels of the website condition, Regul8 with a nurse telephone session and email support, Regul8 with minimal email support, or no website, thus creating 9 groups.

Outcomes: Irritable bowel symptom severity scale and IBS-QOL will be measured at baseline, 6 and 12 weeks as the primary outcomes. An intention to treat analysis will be undertaken by ANCOVA for a factorial trial.

**Discussion:**

This pilot will provide valuable information for a larger trial. Determining the effectiveness of commonly used drug treatments will help patients and doctors make informed treatment decisions regarding drug management of IBS symptoms, enabling better targeting of treatment. A web-based self-management CBT programme for IBS developed in partnership with patients has the potential to benefit large numbers of patients with low cost to the NHS. Assessment of the amount of email or therapist support required for the website will enable economic analysis to be undertaken.

**Trial Registration:**

ClinicalTrials.gov Identifier (NCT number): NCT00934973.

## Background

Irritable bowel syndrome (IBS) is a common chronic gastrointestinal disorder that affects 10 - 22% of the UK population and costs the National Health Service (NHS) over 200 million pounds a year [1,2]. Abdominal pain, bloating and altered bowel habit affect quality of life, social functioning and time off work [3,4]. Treatment relies on a positive diagnosis, reassurance, lifestyle advice, and drug and psychological therapies. However, many patients suffer ongoing symptoms. Drug treatment includes: anti-spasmodics, dietary fibre/bulking agents, antidepressants and anti-diarrhoeals. Bulking agents and antispasmodics and are the most commonly prescribed medications in the UK and Europe. Newer '5HT' antagonist drugs, such as alosetron, have been developed over recent years and used in the U.S.A. but have been hampered by problematic side effects such as ischaemic colitis and severe constipation[5] and are not licenced in the UK. There is a significant psychological aspect to IBS in many patients and psychological therapies, CBT, biofeedback and hypnotherapy can help[6], but availability of these treatments is limited.

A recent Cochrane review [7] highlighted the lack of evidence for the current drug management. Most studies were undertaken a long time ago, are of poor quality, with small numbers, in a secondary care setting. It found benefit for anti-spasmodics for abdominal pain and global assessment of symptoms as a class but said it was unclear whether individual antispasmodic were effective. Only 2 eligible studies [8,9] were found for mebeverine (the most used antispasmodic in the UK) and these failed to show any significant benefit. This may be due to poor design and small numbers. Meta-analysis of the trials on bulking agents also failed to show any significant benefit for in IBS in the Cochrane review[7] or in other published reviews[2,10]. The lack of good quality research into the 'classic' drugs for IBS has also been highlighted in guidelines for IBS, i.e. The British Society of Gastroenterology guidelines for the Management of Irritable Bowel Syndrome[3]. Thus, neither doctors nor patients have good evidence to inform prescribing decisions. However, IBS drugs are recommended in the guidelines [3,11] and widely used. In 2005, NHS costs were nearly £10 million for mebeverine and over £8 million for fibre-based bulking agents (Prescription Cost Analysis figures). A large well conducted trial of mebeverine and a fibre-based bulking agent is needed to provide evidence for prescribing in IBS.

Face to face Cognitive Behavioural Therapy (CBT) has been shown to be helpful for IBS reducing symptom scores and improving QOL measures [6,12] but availability in primary care is limited and CBT in this format has not been found to be cost effective (McCrone P, Knapp M, Kennedy T, Darnley S, Seed P, Jones R *et al*: Cost effectiveness of cognitive behaviour therapy in addition to mebeverine for irritable bowel syndrome, submitted). Additionally there are problems with high drop out rates (McCrone P, Knapp M, Kennedy T, Darnley S, Seed P, Jones R *et al*: Cost effectiveness of cognitive behaviour therapy in addition to mebeverine for irritable bowel syndrome, submitted).

Web-based CBT has been shown to be helpful for other conditions e.g. depression[13] and tinnitus[14]. Thus this could be an efficient, cost effective way of providing help to those with IBS. Despite extensive literature searching before the start of the study, we found no published evaluations of computer-based CBT programmes for IBS. The Gut Trust (formerly called the IBS network) (a patient self-help group) provides an on-line self-management programme (at a charge) but this is not CBT-based and has not been critically evaluated in a trial. NICE has recently recommended the use of Computer CBT for depression, panic and phobia in Primary Care[15]. Development and testing of a computerised CBT programme for IBS has the potential to make CBT more widely available for IBS without increased costs. The increasing availability of the internet makes this a good medium to provide easily accessible patient information and self-management programmes. The majority of households in the UK have internet access (Office for National Statistics August 2009) with figures increasing each year currently 70% have access and this an increase of 11% on the previous year.

### Background to the Project

Over the last year we developed a web-based CBT self-management programme for IBS based on a paper-based manual[16] that was originally developed and tested by one of our team in a RCT of 70 patients in primary care (RMM) [17]. As each module was developed think aloud[18] interviews were undertaken with four patients with IBS whilst they worked through the programme to ensure that the website addressed issues important to people with IBS and was relevant, understandable, navigable and user friendly. The interviews were transcribed verbatim and the feedback was used to modify the site as it was developed.

The website was created using the LifeGuide software [19], which allows people without a programming background to create web-delivered interactive interventions. The web-based intervention we developed consists of 8 sessions for participants to work through over 6 weeks (see table 1 for an overview of each session) and includes interactive components such as: the development of a personal model, creating symptom diaries, goal sheets and thought records. Interactive components help users to remember advice and reflect and provide a "substitute" for the therapist and personalises programme to user [19] This enables users to focus on personally relevant aspects of the programme. Early in each session participants review key points from the previous session and review participant homework. The website includes a 'My Tasks' Section that records participant previous answers and homework.

**Table 1:** Summary of the Regul8 Web-Based Self-Management Sessions

Session 1: Understanding your IBS	Rationale for self-management which includes the following explanations: 1. Possible causes of IBS and illustrative physiology of the digestive system together with the functional changes that occur in the gut as a result of IBS. 2. How the autonomic nervous system ("fight-or-flight" stress system) may interact with the enteric nervous system
**Session 2:** Assessing your symptoms	Self-assessment of the interaction between thoughts, feeling and behaviours and how these can impact on stress levels and gut symptoms. Development of a personal model of IBS which incorporates these elements. Homework: Daily diaries of the severity and experience of IBS symptoms in conjunction with stress levels and eating routines/behaviours

**Session 3**. Managing Symptoms and Eating	Review of the symptom diary Behavioural management of the symptoms of diarrhoea and constipation, and common myths in this area are discussed. Goal setting is explained. The importance of healthy, regular eating and not being overly focused on elimination is covered. Homework: Goal setting for managing symptoms and regular/healthy eating. Goal setting, monitoring and evaluation continue weekly throughout the programme.

**Session 4**. Exercise and Activity	Importance of exercise in symptom management is covered Identifying activity patterns such as resting too much in response to symptoms or an all-or-nothing style of activity is addressed. Homework: Goal setting for regular exercise and managing unhelpful activity patterns if relevant.

**Session 5**. Identifying your thought patterns	Identifying unhelpful thought (negative automatic thoughts) in relation to high personal expectations and IBS symptoms is introduced. Link between these thoughts, feelings, behaviours and symptoms is reinforced. Homework: Goal setting plus daily thought records of unhelpful thoughts related to personal expectations and patterns of over activity.

**Session 6**. Alternative thoughts	The steps for coming up with alternatives to unhelpful thoughts are covered together with personal examples. Homework: Goal setting plus daily thought records including coming up with realistic alternative thoughts.

**Session 7**. Managing Stress and Sleep	Basic stress management and sleep hygiene are discussed. Diaphragmatic breathing, progressive muscle relaxation and guided imagery relaxation are presented in video and audio formats. Homework: Goal setting for stress management, relaxation techniques and good sleep habits.

**Session 8**. Managing flare-ups and the future	The probability of flare-ups is discussed and patients are encouraged to develop achievable, long term goals and to continue to employ the skills they have learnt throughout the manual to manage flare-ups and ongoing symptoms.

### Aims

To pilot a factorial RCT to assess the effectiveness of the prescribed medications in UK general practice for IBS: mebeverine (an anti-spasmodic) and methylcellulose (bulking-agent) against placebo and Regul8, a CBT based self-management website for IBS developed specifically for this study.

The pilot will provide information for a definitive trial and all the trial procedures will be tested. The results of the pilot will enable decisions to be made regarding the full trial. For example:

1) Is it appropriate to test a fibre-based bulking agent in all groups of IBS patient (i.e. in those with diarrhoea symptoms and alternating symptoms as well as those with constipation symptoms)? Most guidelines advocate bulking agents for those with constipation symptoms to increase stool weight and accelerate gut transit [3,11]. The position in those with alternating or diarrhoea symptoms is less certain - increasing stool bulk may stabilise bowel habit and reduce pain or may exacerbate loose stools - trials have tested bulking agents in IBS patients with all 3 patterns of bowel habit and symptom exacerbation seems rare [4,8,20,21]. However, if the results of the pilot show that methylcellulose may significantly worsens symptoms in those with diarrhoea then this group will not be randomised to methylcellulose in the full trial.

2) Should the full trial test the website with minimal email support on request or with the scheduled telephone nurse consultation after session two plus the minimal email support?

## Methods/Design

### Design

Placebo controlled randomised controlled trial with a 3 (drug) × 3 (self-management website) factorial design

### Participants

Inclusion criteria are patients aged 16 to 60 years presenting to general practice with symptoms of IBS that fulfil the Rome III criteria (maximum age 60 years as NICE guidelines advise that a new change in bowel habit in over 60 year olds should have further investigations[11])

### Exclusion criteria

Atypical symptoms - (unexplained weight loss, rectal bleeding), diagnosis of inflammatory bowel disease, Coeliac disease or peptic ulcer disease, pregnant or breast feeding, currently taking or allergy to mebeverine or methylcellulose. If patients are already taking mebeverine or methlycellulose but are keen to enter the trial they will be able to do so if they fulfil the inclusion criteria, do not meet any of the other exclusion criteria and they undertake a 4 week mebeverine and methylcellulose free wash out period before entering the trial.

### Withdrawal criteria

Participants will be withdrawn from the trial if there are any safety concerns regarding continuing the trial medication, if there are any concerns regarding informed consent or if the participant chooses to withdraw.

### Recruitment

Patients will be identified by searching general practitioners' lists for those with a diagnosis of IBS and by opportunistic recruitment of patients presenting with symptoms consistent with IBS. We will utilise the English Primary Care network (PCRN) to aid recruitment and retention of GP practices. We will include practices with urban and rural settings and with a range of socio-demographic characteristics. GP practices willing to participate in the study will search their list for patients aged 16 to 60 with a diagnosis of IBS. Potential participants will be contacted by letter (sent by the GP surgery) informing them about the trial and inviting them to take part. The GPs will check the lists of patients to be contacted prior to the invite letters being sent out to ensure that it is appropriate to contact them. The mailing will include the MIBS patient information sheet. Participants who are interested in participating in the study will return a reply slip with their contact details in a prepaid response envelope to the research team. The reply slip will also enclose a decline sheet where the participants can tick the option that describes the reasons why they have decided to decline the invitation. GPs will also be able to opportunistically provide information about the trial to potential recruits during their GP surgeries. If a patient with IBS attends a GP consultation, GPs will give them the patient information sheet regarding the trial and the reply slip and envelope.

### Intervention

135 patients will be randomised to: mebeverine (135 mg three times a day), methylcellulose (3 tablets twice a day), or a placebo tablet for 6 weeks. To ensure double blinding, all participants will take three over-encapsulated identical tablets in the morning, one at lunchtime and three at teatime.

They will also be randomised to 1 of 3 website conditions: access to Regul8 with a 30-45 minute nurse telephone session after session 2 (to encourage engagement with the CBT programme) and email support on request, the website with minimal support (i.e. technical email support on request), or no website, thus creating 9 groups.

There will be double-blinding for medication groups, single-blinding for the website (participants will not be able to be blind to website access). Website access will be with a personal password so that usage can be assessed. All patients will receive a telephone call in the first week to check they have no problems with the medication or paperwork. Those not randomised to website access will receive standard patient information and will be offered access to the website at the end of the trial.

### Telephone session

The telephone session in the first website condition will be scheduled after patients complete session 2 where they develop their own personal model on the website of how their thoughts, feelings and behaviours might be contributing to their IBS symptoms. The purpose of the session is for the patients to clarify their model with the nurse, for the nurse to help patients extend their model where they may have left out relevant material, and to motivate patients to continue using the website by linking the sessions back to the information they provide. Patients will also be given the opportunity to discuss any technical difficulties they may have and encouraged to email the nurse if they wish to clarify any information on the site or have any difficulties in the future.

Prior to the start of the trial the nurse received 4 hours training in the telephone protocol which included outlining the protocol, training in the basic principles of CBT and listening skills, practice role play sessions which were audiotaped and discussed in supervision sessions with RMM. All sessions will be audiotaped and saved in password protected anonymous files. Throughout the trial, audiotapes will be sent to RMM and the nurse will receive regular supervision (after each session for the first 4 sessions and 4 random sessions thereafter. This will ensure the quality the intervention as well as allow us to assess fidelity regarding the protocol. The timing and length of each telephone session will be recorded, as well as the number of email contacts per participant both in the telephone and email only conditions.

### Trial medication

Will be over-encapsulated active mebeverine 135 mg tablets, over-encapsulated active methylcellulose 500 mg tablets and over-encapsulated placebo (candarel) tablets all to be taken orally. All patients will receive medications of the same shape and colour. The patients will be supplied with 6 weeks of medication by the research nurse. To ensure double blinding, all participants will take 3 over-encapsulated identical tablets in the morning, one at lunchtime and three at teatime.

The medications will be supplied by SCM Pharma and fully QP released in line with MHRA regulations and cGMP procedures. SCM pharma will produce all patient kits according to the randomisation list and will supply code break envelopes to ensure patient safety.

The medications are being supplied in two packing campaigns, with a shelf life of 12 months. The study medications will be stored and dispensed by the trials site pharmacy in accordance with Good Clinical Practice and Good Manufacturing Guidelines. All the participants will receive a Medication Diary Sheet where they will keep a daily record of the numbers of tablets taken during the six week period.

### Randomisation

A randomisation list will be computer generated by the trial statistician (PS). Participants will be block randomised in blocks of 9 and will be stratified by type of IBS (i.e. diarrhoea predominant, constipation predominant, alternating- pattern) as there may be a different response to the medications between these sub-groups. The Company who are supplying the trial medications will make up the patient packs according to a randomisation list supplied by the statistician so that the research nurse is not aware of which trial medication the participant receives. At randomisation, the research nurse gives the participant the next sequential numbered pack from the appropriate strata of IBS type for that participant's symptoms.

### Data collection

Participant data will be collected on the MIBS trial website. Baseline data will be collected from included patients before randomisation and will be co-ordinated by the trial manager who will be blind to treatment allocation. All baseline and outcome data is patient self-completed away from the study team, thus avoiding any influence of the study team on the responses and reducing bias. Participants will be given a unique password to log onto the website. Their data will be identified by a unique identification number and will be kept separate from any personal identifying data to maintain confidentiality.

An on-line baseline assessment which includes socio-demographic details and the measures detailed below will be completed by all participants prior to the nurse visit and before randomisation. These are well-recognised, validated measures that are widely used and will enable us to gain a clear picture of participants IBS and the impact it has on their lives.

Those randomised to the self management programme will complete on-line symptom severity questions, symptom, food and exercise diaries, questions about stress and triggers and their symptoms and their coping strategies. These data will be collected and stored and may be used to provide information to assess the self management programme but will mostly be used by the participants themselves to monitor their progress and inform themselves about their own symptoms, triggers and coping strategies as part of the cognitive behavioural therapy based self-management programme.

Outcome data and questionnaires outlined below will be completed at 6 and 12 weeks post randomisation by all participants. Participants will be sent a reminder email at 6 and 12 weeks to prompt them to complete the questionnaires. If it has not been completed within one week of the reminder, a further reminder will be sent. One week after that, if no data has been entered, the research nurse will ring the participant to ask if she can collect the data over the telephone.

The patients GP notes will be reviewed at 12 weeks to assess the number of GP contacts in the year prior to entering the study and in the 3 months since the study. In a full trial a one year notes review is planned. Other studies have shown an impact on GP contacts from patient self management programmes[22].

### Study Procedures: (See Additional File 1)

Those responding to the recruitment letter from their GP will be asked to complete a screening on-line questionnaire consisting of the Rome III criteria and questions about exclusion and inclusion criteria to check if they fulfil the eligibility criteria for the study. They will log on to the MIBS trial website with a unique ID number. Any patient indicating they may have a 'red flag' symptom that would indicate the need for further investigations (i.e. unexplained weight loss or rectal bleeding) will be referred back to their GP and would not enter the study.

Those who are eligible to enter the trial will be telephoned by the research nurse and offered a meeting with the research nurse at their GP practice to discuss the trial and complete consent forms. At this meeting a blood sample will be taken from the participant by the research nurse for a full blood count (FBC), transglutaminase antibodies (TTG) and a C-Reactive protein (CRP) to exclude alternative diagnoses i.e. anaemia that requires further investigation and Coeliac disease. The results will be made available to the participants GP. The blood sample will not be stored for future use.

Once the blood test result is known and the patients eligibility for the trial is confirmed (i.e. no abnormalities on the blood test) a further nurse appointment will be made and the participant will be given a recruitment pack containing their trial medication and an information sheet about the website and website password, or written patient information on IBS. If the blood tests show an abnormal result i.e. an CRP over the normal laboratory range or anaemia or a positive test for Coeliac disease the patient will not be randomised to the trial but will be referred back the their GP for further assessment.

The research nurse will telephone all participants in the trial in the first week after randomisation (see randomisation procedure above) to check they have no problems with the medication or paperwork and to ensure they can log onto the MIBS website. The nurse will also make a time for a phone call session with those randomised to the Regul8 self-management programme with nurse support (see section above for details on telephone session protocol and timing). They will also be able to email the nurse regarding queries about the website programme during the study. Medical questions will not be addressed by the research nurse and participants will be advised to seek medical advice if they have medical queries. Those randomised to website alone will be offered technical website support by email.

Those not randomised to receive the website programme will receive normal GP care. All the randomised participants will receive normal GP care during the trial if they require it.

The trial medication for the MIBS Study is over-encapsulated to maintain blinding but is quite large and preliminary work indicated that some participants may find it difficult to swallow. If participants were considering withdrawal from the trial due to the size of the medication and swallowing problems, they will be permitted to open the medication capsules to enable them to continue to take the medication and stay within the trial. The Research Nurse (RN) will discuss medication taking with any participants who indicate that they are finding it difficult to take. If they indicate that they cannot manage to take the trial medication because of swallowing problems with the capsules, the RN will provide the option that they could open the capsules by twisting them and taking the medication inside the capsule. This is smaller than the entire capsule and thus would mean the medication is easier to swallow and should enable retention of these participants within the study.

These participants will potentially be un-blinded as they will see the tablets. However, participants will be recording their medication taking in diaries and will be asked to guess which medication they are on - enabling assessment as to whether opening the capsule un-blinds the participants. We will record which participants open the capsules. Intention to treat analysis will be maintained but will include the opening of the capsules as factor within the analysis of the data.

### Primary Outcome measures

Change in the Irritable Bowel Symptom Severity Scale (IBS SSS score) and IBS Quality of Life Questionnaire (IBS-QOL) from baseline to 12 weeks.

The IBS SSS [23] is a 5 item self-administered questionnaire measuring: severity of abdominal pain, duration of abdominal pain, abdominal distension/tightness, bowel habit, quality of life. Maximum score 500: < 75 normal bowel function, 75-174 mild IBS, 175-299 moderate IBS, 300-500 severe IBS). The IBS-QOL[24] is an extensively validated quality of life measure for IBS: 34 items, 8 subscales, item responses graded on a 5 point scale, total score 0-100 with higher scores indicating better health related QOL. Administered at baseline and 6 weeks (end of treatment) and12 weeks (6 months and 12 months would also be planned for the full trial).

### Secondary outcome measures

The Subjects Global Assessment of Relief (SGA of Relief)[25] is frequently used in treatment trials to identify IBS responders to therapy. Participants rate their relief from IBS symptoms on a scale of 1 to 5 ranging from "completely relieved" to "worse". Scores are dichotomized so that patients scoring from 1-3 are considered responders and those 4-5, non-responders.

The Hospital Anxiety and Depression Scale (HADS)[26] is a commonly used self-report instrument for detecting depression and anxiety in patients with medical illnesses. It enables assessment of participants' level of anxiety and depression which has shown to be high in many patients with IBS.

Patient Enablement Questionnaire[27] assesses participants' ability to cope with their illness and life. The number of Primary Care consultations for IBS will also be assessed to look for any change. Other studies have shown an impact on GP contacts from patient self management programmes [22].

The acceptability of the Regul8 self-management treatment will be assessed using three questions where patients rate the overall effectiveness of the programme, the efficacy of programme compared to other treatments they have tried, and whether they enjoyed the programme.

Qualitative interviews will also be undertaken at the end of treatment with a small number of the participants regarding their experience of participating in the trial and the acceptability of the trial procedures, including the medication taking. This will provide richer and more detailed information on the trial experience and provide valuable information to inform a full trial.

Up to 30 participants will be invited to participate, using purposive sampling to ensure we sample people with a range of age, gender, symptoms and treatment combinations. The interviews will be semi-structured with open ended questions covering the following topics: Main reasons to try out the treatment; Expectations of the treatment before initially trying it; Positive and negative perceptions of the treatment; Changes during and after the end of treatment; Looking back at the treatment (thoughts and feelings). The set of generic questions will be repeated for each treatment type that is mentioned by the participants and for the intervention received during the trial.

All interviews will be tape-recorded and full transcribed, and inductive (data driven) thematic analysis will be used to analyse the data following the methods described by Boyatzis (1998)[28], Braun and Clarke (2006)[29], and Joffe and Yardley (2004)[30].

### Sample size

The aim of the pilot is to have sufficient numbers to test the feasibility of the trial and assess crude differences between the groups. Assuming an initial mean symptom score of 250 (SD 80) in all groups, 35 patients are required in each group to detect a difference in score of 53 points (2/3 of a SD of 80) between the treatment groups (mebeverine, methylcellulose and placebo) or the three website groups (none, minimal support, telephone) for 80% power and 95% confidence. Allowing for drop outs at 20%, 130 participants would be needed. If 135 are recruited each of the 9 randomisation groups would potentially have 15 recruits.

For IBS-QOL, 34 would be required in each group to detect a 10 point change in the IBS-QOL in the intervention group assuming a score in the placebo group of 60 (SD of 15). Hence, allowing for a 20% drop-out rate, again 135 participants would be more than sufficient.

### Basis of assumptions for power calculation

The Kennedy[12] paper (2005) using the symptom score in general practices in London found a baseline symptom score of approximately 300 (SD 80) in the IBS SSS in their trial of cognitive behavioural therapy and mebeverine and a treatment effect of a 68 point reduction for CBT and mebeverine together over mebeverine alone at 6 weeks after the CBT. (Their a priori power calculation was on the basis of a score of 180 (SD80) in the control group and 133 (SD80) in the CBT (treatment) group at 6 months follow up.)

The IBS-QOL overall score was found to be 63.2 (SD 18.5) in validation scores for the measure[24], and a pre-treatment score of 65.8 (SD19.9) with a post-treatment score 10.2 points higher was found in further validation of the measure in female patients with functional bowel disorder in secondary care assessing cognitive behavioural or patient education versus desipramine or placebo[31].

### Analysis

An intention-to-treat analysis, of change in the IBS SSS and IBS-QOL at baseline, 6 weeks and 12 weeks (6 mnth and 1 yr are planned for the full trial) will be undertaken by ANCOVA for a factorial trial. In the main trial (but not in the pilot): subgroup analysis will be performed using regression modelling to determine if symptoms subgroup (diarrhoea-predominate, constipation-predominate and alternating symptoms) predicts differences in outcome. Other potential factors that may affect outcome will be explored: severity of presenting symptoms, duration of presenting symptoms, age and sex.

### Ethical approval

The study has been reviewed and approved by Southampton Research Ethics Committee A number: 09/H0502/101

### MHRA approval

The Study has been reviewed and approved by the MHRA. EudraCT number: 2009-013426-16

### Trial Sponsorship

Provided by the University of Southampton

### Trial Funder

Research for Patient Benefit Programme, National Institute of Health Research, NHS, UK

### Safety Reporting

Adverse events will be recorded in accordance with the University of Southampton Research Related Adverse Event Reporting Policy and with the European Directive 2001/20/EC.

### Monitoring and Audit

The study will be monitored and audited in accordance with Southampton University procedures. All trial related documents will be made available on request for monitoring and audit by the University of Southampton, the relevant REC and for inspection by the MHRA or other licensing bodies.

### Data Protection

Data will be collected and retained in accordance with the Data Protection Act 1998.

### Storage of Records

Study documents (paper and electronic) will be retained in a secure location during and after the trial has finished. All source documents will be retained for a period of 5 years following the end of the study.

### Indemnity

This is an NHS-sponsored research study. For NHS sponsored research HSG(96)48 reference no. 2 refers. If there is negligent harm during the clinical trial when the NHS body owes a duty of care to the person harmed, NHS Indemnity covers NHS staff, medical academic staff with honorary contracts, and those conducting the trial. NHS Indemnity does not offer no-fault compensation and is unable to agree in advance to pay compensation for non-negligent harm. Ex-gratia payments may be considered in the case of a claim.

A Steering committee, including a patient representative, will oversee the trial procedures and ensure good conduct of the study; they will meet at least annually.

### The roles and responsibilities of each member of the team

HE will be principal investigator and oversee the running of the study with support from PL. LY will oversee the website development, RMM will oversee adaptation of the CBT programme to a web-based format and supervision of the nurse delivered intervention.

Regular updates and meetings will ensure good communication. The collaborators will meet at least 4 times a year. The research assistant will circulate a monthly update to review progress relative to the project plan, highlighting any issues that need to be addressed. Each team member will consult the other team members immediately by email and/or phone on any issues that arise.

### Trial status

The trial opened to recruitment in April 2010 and recruitment and follow up will continue until February 2011. Results will be analysed and reported in 2011.

## Discussion

IBS is a common problem managed largely in general practice. Many patients live with significant restrictions on their daily activities and reduced quality of life. There is a lack of research evidence on the commonly prescribed medications and a lack of availability of psychological therapies such as CBT which have been shown to be beneficial in IBS. Provision of a web-based CBT self-management programme for IBS patients has the potential to open up new resources for IBS patients with minimal additional cost in an area where there is significant ill-health burden. Clarifying the effectiveness of commonly prescribed medications will allow better informed prescribing decisions and targeting of NHS resources.

This pilot study is the first to look at providing CBT as a web-based self-management programme for patients with IBS. The factorial design allows the effectiveness of common IBS medications to be assessed at the same time, maximising the amount of data collected. The trial will answer important questions regarding the feasibility of undertaking this type of research, which requires the involvement of large numbers of patients from many GP practices across different PCTs. Trial systems will be tested and data will be collected about recruitment and drop-out rates, reasons for declining to take part, acceptability of the medication and of the outcome measures. Data on the variance and estimated effects of the intervention will also be able to be assessed. It will also provide guidance on the amount of support/therapist time that is likely to be required for the self-management programme. The qualitative data will highlight useful changes in the self-management programme or the study design for a larger trial. This is a pilot study and the feasibility issues will be the main useful outcomes as the trial is not powered to detect a definitive effects size. It will however, have power to demonstrate whether there are moderate to large effects.

The trial methods comply with best practice for the conduct and reporting of RCTs and thus will provide good quality data and will be able to be replicated on a larger scale.

## Competing interests

The authors declare that they have no competing interests.

## Authors' contributions

HE, RM, PL and LY were involved in the conception and design of the study and applied for funding. HE wrote the first draft of the grant application, is Lead investigator and Principal Investigator. RMM oversaw the adaptation of the paper-based CBT manual into the website, the development of the telephone protocol, and nurse training and supervision. LT and AS developed the website and managed the trial. LY oversaw the website programming package. NC (Consultant Gastroenterologist) was a clinical expert advisor. PS developed the Statistical Plan and Randomisation. All Authors read and approved the final manuscript.

## Pre-publication history

The pre-publication history for this paper can be accessed here:

http://www.biomedcentral.com/1471-230X/10/136/prepub

## Supplementary Material

Additional File 1 **Patient flow and data management diagram**. This diagram is a detailed summary of the main steps and procedures of the trial following a chronological order. Click here for file
