# Supplementary material for: Management of irritable bowel syndrome in primary care: feasibility randomised controlled trial of mebeverine, methylcellulose, placebo and a patient self-management cognitive behavioural therapy website. (MIBS trial)
Source: BMC Gastroenterol. 2010 Nov 18;10:136. doi: 10.1186/1471-230X-10-136 (PMC2998449; doi:10.1186/1471-230X-10-136)
Supplement: Additional File 1 — Patient flow and data management diagram. This diagram is a detailed summary of the main steps and procedures of the trial following a chronological order. [file 1471-230X-10-136-S1.DOC]

Additional Files

Additional File 1

Title: Patient flow and data management diagram

Description: This diagram is a detailed summary of the main steps and procedures of the trial following a chronological order

Patient receives GP letter

Returns letter to trial manager TM

TM emails patient website link and asks them to sign up and do screening q

Patient completes screening questionnaire

Problematic exclusion: Trial manager sent email if participant Sec B Q1(lost weight) or Q8 (rectal bleeding) = yes

Included: Patient receives automated response on screening

Normal exclusion: Info on NICE guidance

Patient sent letter and told to see GP. GP sent letter also

RN receives automated email providing patient username details and eligibility

TM enters patient contact details onto Google spreadsheet (Trial database).

RN checks trial database to get contact details and arranges appointment.

RN meets patient at GP surgery and takes blood and consent

Bloods sent to General Hospital lab for analysis

RN: Trial database to note bloods taken and consent given

Bloods sent back to RN at Primary Care

RN arranges meeting with patient

SAE recorded and reported to tsc

TM records this in trial database

Patient randomised by to condition

Patient meets with RN. Patient opens pack and RN makes a note of website condition on separate database. RN logs onto website (make sure signed up first) using nurse admin username. RN types in patients username (email) to give them access to the Regul-8 programme

Automated weekly reminders for website

IF SAE occurs, patient/GP contacts unbliniding service and completes SAE form

Form faxed to PI

Patient logs on and completes baseline questionnaire

Email sent for patient log in to 6 and 12 weeks: Outcome questionnaires

Completed

Not completed

RN automated email when 12 week completed and notifies RN to arrange medicine pick up. Completes drug log

Auto reminders sent out and RN notified by automatic email

If still no response – call from RN

If patient in website condition – must complete programme before logging onto 6 week outcome q

RN: Logs onto management enables eligible patients = access to baseline questionnaire

If SUSAR, TM notifies ethics committee, TSC and sponsor and funder.

RN picks up medication pack from primary care.

If telephone session – RN arranges appt and notes on separate database

RN completes drug log

Technical prob – RN receives email and forwards to TM
